# Linc00462 promotes pancreatic cancer invasiveness through the miR-665/TGFBR1-TGFBR2/SMAD2/3 pathway

**DOI:** 10.1038/s41419-018-0724-5

**Published:** 2018-06-13

**Authors:** Bin Zhou, Weidong Guo, Chuandong Sun, Bingyuan Zhang, Fang Zheng

**Affiliations:** 1 Department of Hepatopacreatobiliary, Affiliated Hospital of Qingdao University, 266003 Qingdao, China; 2 School of Integrative Medicine, Tianjin Traditional Medical University, 300193 Tianjin, China

## Abstract

Emerging evidence has identified that long non-coding RNAs (lncRNAs) may play an important role in the pathogenesis of many cancers, pancreatic cancer (PC) included. However, the role of linc00462 in PC remains unclear. The aim of our present study was to investigate the potential functions of linc00462 in PC and to identify the underlying mechanisms of action. CCK8 assay, transwell assay, cell cycle assay, cell apoptosis assay, EdU assay, western blot assay, cell adhesion assay, HE staining, IF staining, ELISA assay, vivo growth and metastasis assay, and colony formation assay were performed. We demonstrated that OSM mediated up-regulation of linc00462 promoted cell proliferation by accelerating cell cycle process and inhibiting cell apoptosis and adhesion in vitro, enhanced cell migration and invasion by accelerating EMT process, promoted tumor growth and matastasis in vivo and was associated with large tumor size, poor tumor differentiation, TNM stage and distant metastasis in patients of PC. In addition, we demonstrated that linc00462 was a target of miR-665. Linc00462 overexpression enhanced the expression levels of TGFBR1 and TGFBR2, and thus activated the SMAD2/3 pathway in PC cells. In conclusion, linc00462/miR-665/TGFBR1/2 regulatory network may shed light on tumorigenesis in PC.

## Introduction

Pancreatic cancer (PC) is one of the most commonly diagnosed cancers and there have been few advances in treatment in the past decades^1^. For many years, Gemcitabine was the only drug approved to treat this malignant disease^2^. However, the resistance of pancreatic cancer cells to Gemcitabine occurs repeatedly in patients during the process of treatment and is identified as one of the major reason for cancer progression^3^. Furthermore, the epithelial-mesenchymal transition (EMT) in vitro and metastasis in vivo are closely involved with the pathogenesis and progression of PC^4–6^. More importantly, there are neither validated predictive nor prognostic biomarkers for this lethal disease. Thus, it is imperative to investigate the molecular mechanism underlying the development and progression of PC and explore the targeted signaling pathways for cancer treatment.

Long non-coding RNAs (lncRNAs) are RNA molecules over 200 nt in length that do not encode proteins^7,8^. Recent studies have revealed that lncRNAs are involved in gene regulation and various aspects of tumor cellular homeostasis, including tumor growth, development, differentiation, proliferation, apoptosis and metastasis^7,9,10^. For example, up-regulation of linc00673 promoted cell proliferation, cell migration, cell invasion and EMT in non-small cell lung cancer^11^. In pancreatic cancer, data also demonstrated that some differentially regulated lncRNAs are correlated with malignant phenotype and prognosis in patients^12–15^. For example, lncRNA TUG1 enhanced the proliferation and migration of pancreatic cancer cells through EMT pathway^16^. In addition, knock-down of HOTAIR suppressed tumor growth and also reduced the expression of notch3 in pancreatic cancer^17^. Gong et al. reported that linc00462 was significantly upregulated in HCC tissues and overexpression of linc00462 resulted in a much more aggressive oncogenic phenotype via activing the PI3K/AKT signaling pathwayin HCC cells^18^. However, the expression level and biological function of linc00462 in PC still remains unknown.

Various molecular mechanisms of lncRNA underlying cancer development have been proposed^19^. One of the important mechanisms is that the lncRNA acts as a miRNA sponge to regulate the miRNA expression, which inturn regulates the miRNA target genes indirectly^20^. For example, long non-coding RNA X-inactive specific transcript (XIST) is involved in the development and progression of PC through the miR-133a/EGFR pathway^21^. Thus the investigation on whether linc00462 regulating the development and progression of PC and acting as a ceRNA seems to be promising.

In the present study, we identified the oncogenic role of linc00462 which may function as an effective invasiveness marker for PC patients. We found that miR-655 was a potential target of linc00462 by using the bioinformatics software of RegRNA 2.0. We then explored the role of miR-655 in PC cells, which demonstrated the tumor suppressive role of miR-665 via targeting TGFBR1 and TGFBR2 by regulating SMAD2/SMAD3 pathway. Therefore, our results may provide a new insight into understanding the network of linc00462/miR-665/TGFBR1/TGFBR2 in PC and this discovery also provides atheoretical basis for the prevention and treatment for PC.

## Results

### Linc00462 is high expression in PC and is upregulated by OSM in PC cells

**Fig. 1:**
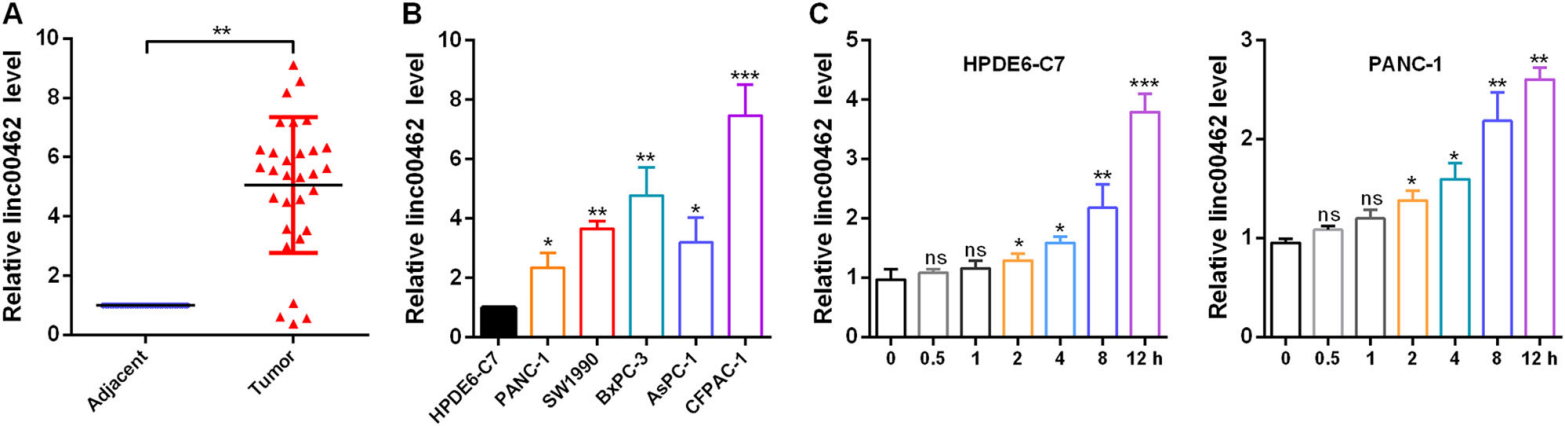
linc00462 is upregulated in PC tissues and cells. **a** The levels of linc00462 in PC and the adjacent normal tissues were examined by RT-qPCR assay. **b** The levels of linc00462 in the indicated cells were examined by RT-qPCR assay. **c** The levels of linc00462 in cells treated with OSM at the indicated time were examined by RT-qPCR assay. All results performed above are presented as mean ± SD from three independent experiments. *p < 0.05; **p < 0.01;***p < 0.001, NS not significant

To confirm the expression level of linc00462, we detected the linc00462 level in 35 paired PC tissues and the adjacent pancreatic tissues. As shown in Fig. 1a, the expression level of linc00462 was significantly higher in tumor tissues (Fig. 1a), which is correlated with large tumor size, poor tumor differentiation, TNM stage and distant metastasis in patients with pancreatic cancer (Table 1). In addition, we examined the expression level of linc00462 in five PC cell lines (PANC-1, SW1990, BxPC-3, AsPC-1, and CFPAC-1) and a normal human pancreatic normal pancreatic epithelial cell line HPDE6-C7. Compared with the HPDE6-C7 cells, PC cells exhibited significantly higher expression level of linc00462 (Fig. 1b). Furthermore, Smigiel has been reported that OSM could regulate an EMT/CSC plasticity program that promotes tumorigenic properties in PC^22^. We then examined whether OSM regulate the expression level of linc00462 in PC cells. As shown in Fig. 1c, the expression level of linc00462 was markedly elevated under OSM treated at different time in HPDE6-C7 and PANC-1 cells (Fig. 1c). Based on the results of the expression level of linc00462, we hypothesized that linc00462 re-expression might promote cell proliferation. PANC-1 and CFPAC-1 were chosen for subsequent functional studies, because of the lowest or highest expression level of linc00462.

**Table1:** Correlation between linc00462 expression and clinical features (*n* = 35)

Variable	linc00462 expression		*p*-value
	Low	High	
*Age*			
<60	11	8	
≥60	6	10	0.194
*Gender*			
Male	14	10	
Female	3	8	0.089
*Tumor size*			
<3	10	3	
≥3	7	15	0.012^a^
*Histological grade*			
High	12	4	
Low	3	14	0.007^a^
*TNM stage*			
I–II	14	2	
III–IV	3	16	0.001^a^
*Distant metastasis*			
Postive	5	17	
Negetive	12	1	0.001^a^

a*χ*^2^ test. *p*-values in bold print indicate statistically significant differences

*TNM* tumor node metastasis

^a^*p* < 0.05

### Linc00462 promotes cell proliferation in PC cells

**Fig. 2:**
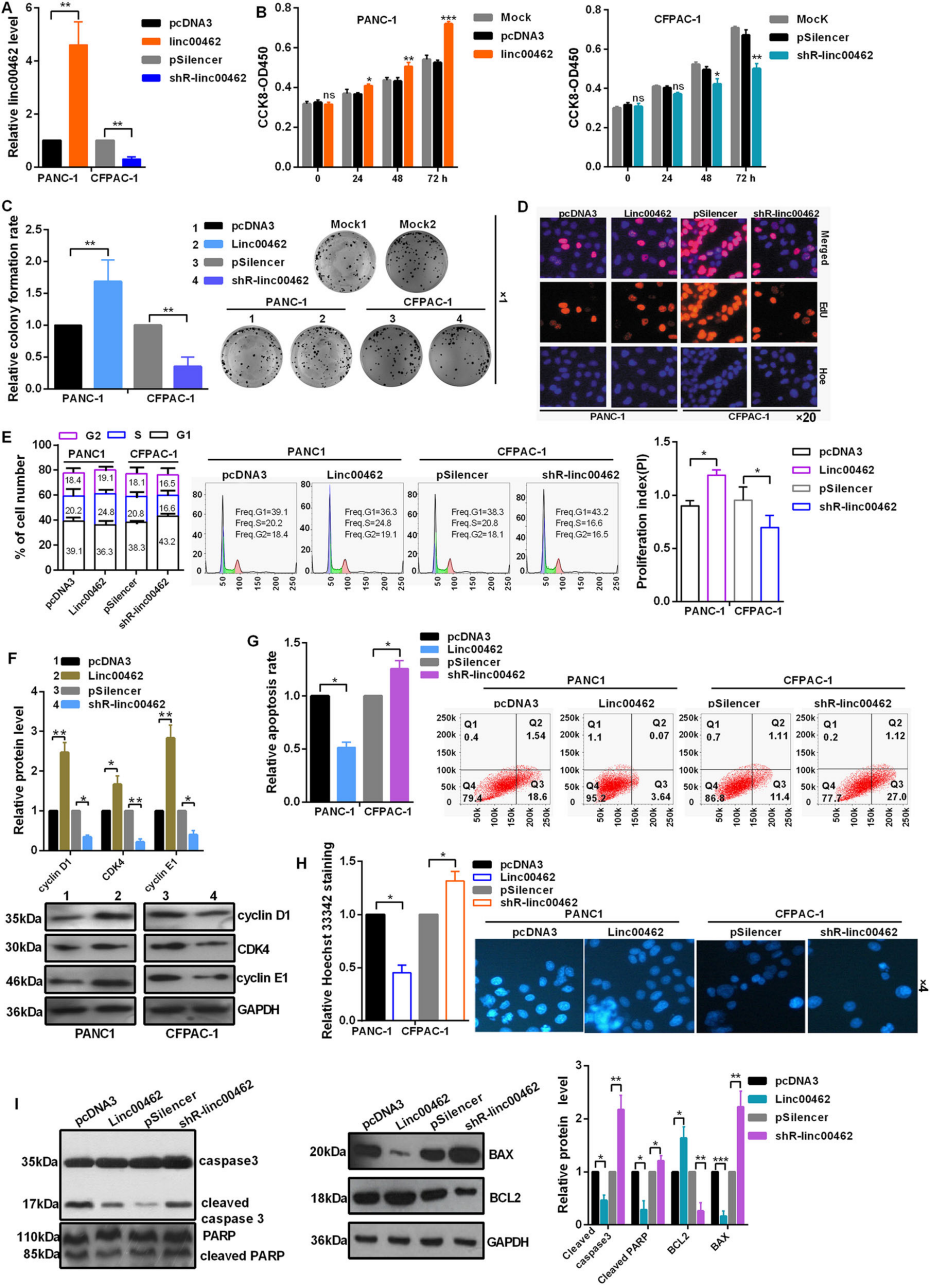
linc00462 promotes cell proliferation in PC cells. **a** The efficiency of the plasmids of linc00462 was identified by RT-qPCR assay. **b** CCK8 asssay showed the effect of linc00462 on cellular viabilities. **c** Colony formation assay showed linc00462 overexpression promoted relative colony formation rate in PC cells. **d** EdU incorporation assays showed that linc00462 promoted the cell numbers of EdU staining. **e** The cell cycle of linc00462 was detected by flow cytometry assay. **f** The related protein levels of cell cycle were detected by western blot assay. **g** Cell apoptosis assays showed that linc00462 overexpression inhibited the apoptosis by flow cytometry assay. **h** Hoechst/PI staining assays showed that linc00462 overexpression inhibited the apoptosis. **i** The related protein levels of cell apoptosis were detected by western blot assay. All results performed above are presented as mean ± SD from three independent experiments. *p < 0.05; **p < 0.01; ***p < 0.001, NS not significant

Transfection efficiency of the plasmids of overexpression and knockdown for linc00462 was detected by RT-qPCR assay (Fig. 2a). CCK-8 and colony formation assay showed that overexpression of linc00462 obviously promoted cell proliferation in PANC-1 cells; On the contrary, linc00462 knockdown significantly reduced the proliferation ability of the CFPAC-1 cells (Fig 2b, c). Moreover, EdU proliferation assays showed that linc00462 overexpression increased EdU incorporated cell proportion and linc00462 knockdown reduced EdU incorporated cell proportion (Fig 2d). In the aspect of cell cycle, overexpression of linc00462 obviously decreased the cell population of G0/G1 phase accompanied by an increase of cell population in the S + G2 phase in PANC-1 cells; knockdown of linc00462 increased the number of G0/G1 phase in CFPAC-1 cells and decreased the cells in S + G2 phase; In addition, linc00462 overexpression promoted proliferation index (PI, PI = (G2 + S)/G1), linc00462 knockdown decreased proliferation index (Fig 2e). Furthermore, we found that overexpression of linc00462 markedly promoted cyclin D1, CDK4 and cyclin E1 expression level in PC cells using western blot assay, which were the important regulators of cell cycle (Fig 2f). Cell apoptosis analysis showed a lower apoptosis rate when linc00462 was overexpressed in PANC-1 cells and a higher apoptosis rate under the knockdown of linc00462 in CFPAC-1 cells (Fig. 2g). In addition, Hoechst staining also demonstrated that linc00462 overexpression reduced cell apoptosis in PANC-1 cells and linc00462 knockdown promoted cell apoptosis in CFPAC-1 cells (Fig 2h). Consistently, the protein levels of cleaved caspase 3 and PARP and BAX were decreased while the levels of BCL2 were increased after linc00462 overexpression in PANC-1 cells (Fig 2i).

### Upregulation of linc00462 facilitates the cell migration, invasion, EMT, and tumor growth and metastasis and inhibits the cell adhesion

**Fig. 3:**
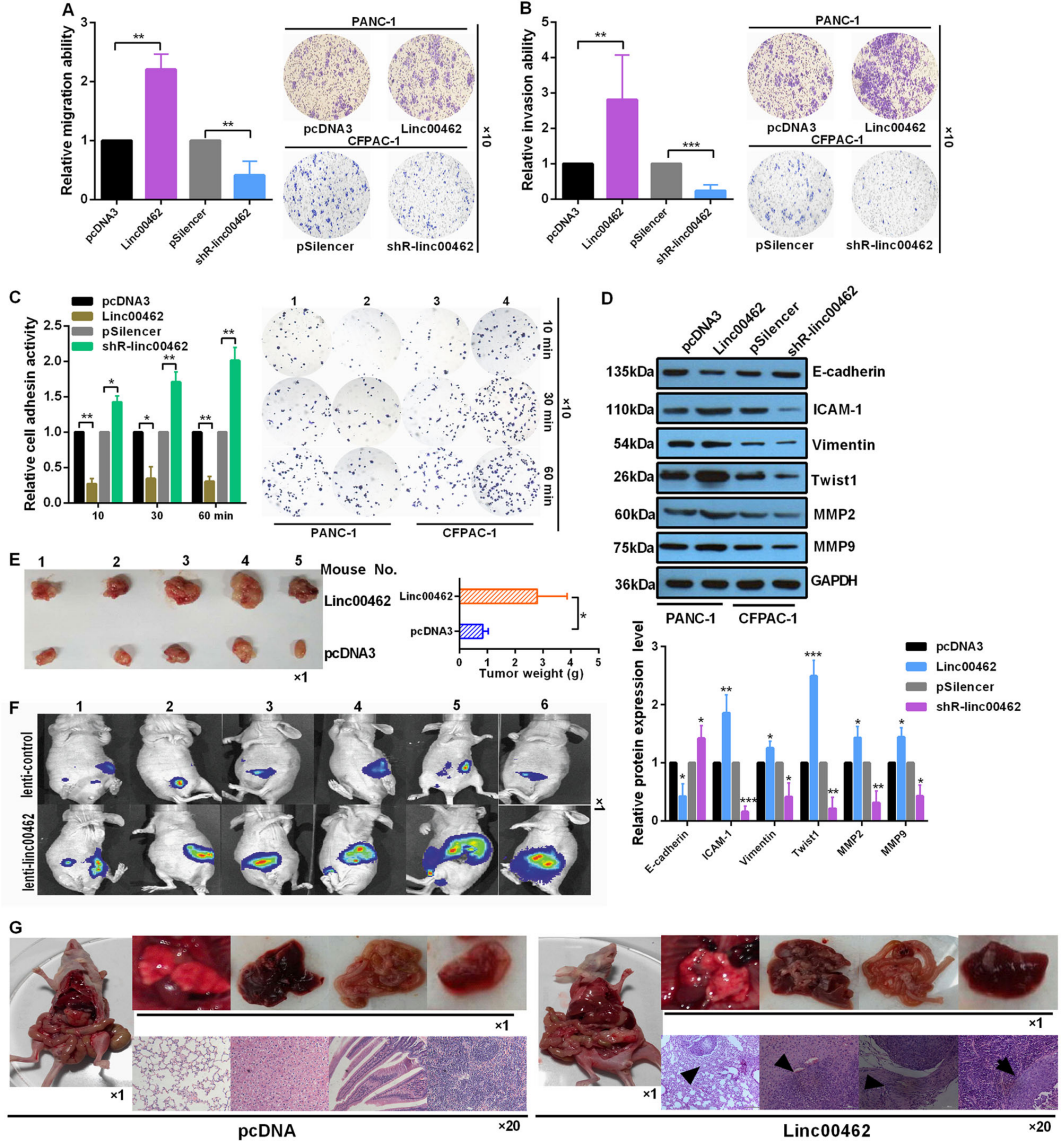
linc00462 promoted EMT and tumor growth, inhibited cell-matrix adhersion. **a**, **b** Transwell assay showed the effect of linc00462 on cell migration and invasion ability. **c** Adhesion assay of PC cells transfected with the indicated plasmids at the indicated time. The number of adherent cells was estimated by reading the absorbance at 620 nm. The photomicrographs were taken at ×100 magnification. **d** The related protein levels of EMT process were detected by western blot assay. **e**, **f** The tumor size and weight were significantly increased after treated with lenti-linc00462. **g** Comparison of spontaneous metastasis and images of representative metastatic foci in the lung, liver, colorectum and spleen by HE staining. All results performed above are presented as mean ± SD from three independent experiments. *p < 0.05; **p < 0.01; ***p < 0.001, NS not significant

The migration and invasion ability of PC cells were determined by transwell migration and invasion assay, respectively. Results showed that the number of migrated and invaded cells was significantly increased treated with linc00462 in PANC-1 cells compared to the group transfected with pcDNA3 (Fig. 3a, b). The cell-matrix adhesion assay indicated that adhesion activities of PANC-1 cells treated with linc00462 were inhibited compared with the control groups, while linc00462 knockdown in CFPAC-1 cells enhanced cell adhesion ability (Fig. 3c). To investigate whether linc00462 could regulate EMT, PC cells were transfected with linc00462 overexpression or knockdown plasmids. Interestingly, western blot analysis showed that overexpression of linc00462 in PANC-1 cells increased the expression level of the molecular marker of mesenchymal cells (ICAM-1, Vimentin, Twist 1, MMP2, and MMP9) and decreased the expression level of E-cadherin, which is the marker of epithelial cells; linc00462 knockdown in CFPAC-1 cells has the opposite effects (Fig. 3d). In vivo, the average weight of tumors increased in the linc00462 treated group compared to the negative control group (Fig. 3e, f). Furthermore, we examined the effects of linc00462 on tumor metastasis using the linc00462 overexpression cell lines and the results showed that the mice injected with PANC-1-linc00462 cells metastasized more efficiently than that injected with the PANC-1-pcDNA3 group. Subsequently, the lung, liver, colorectum and spleen sections were prepared and stained with hematoxylin andeosin (HE), and we detected bigger and more metastatic foci in the samples from mice injected with PANC-1-linc00462 cells than that with control cells (Fig. 3g).

### Linc00462 acts as a molecular sponge for miR-665 in PC cells

**Fig. 4:**
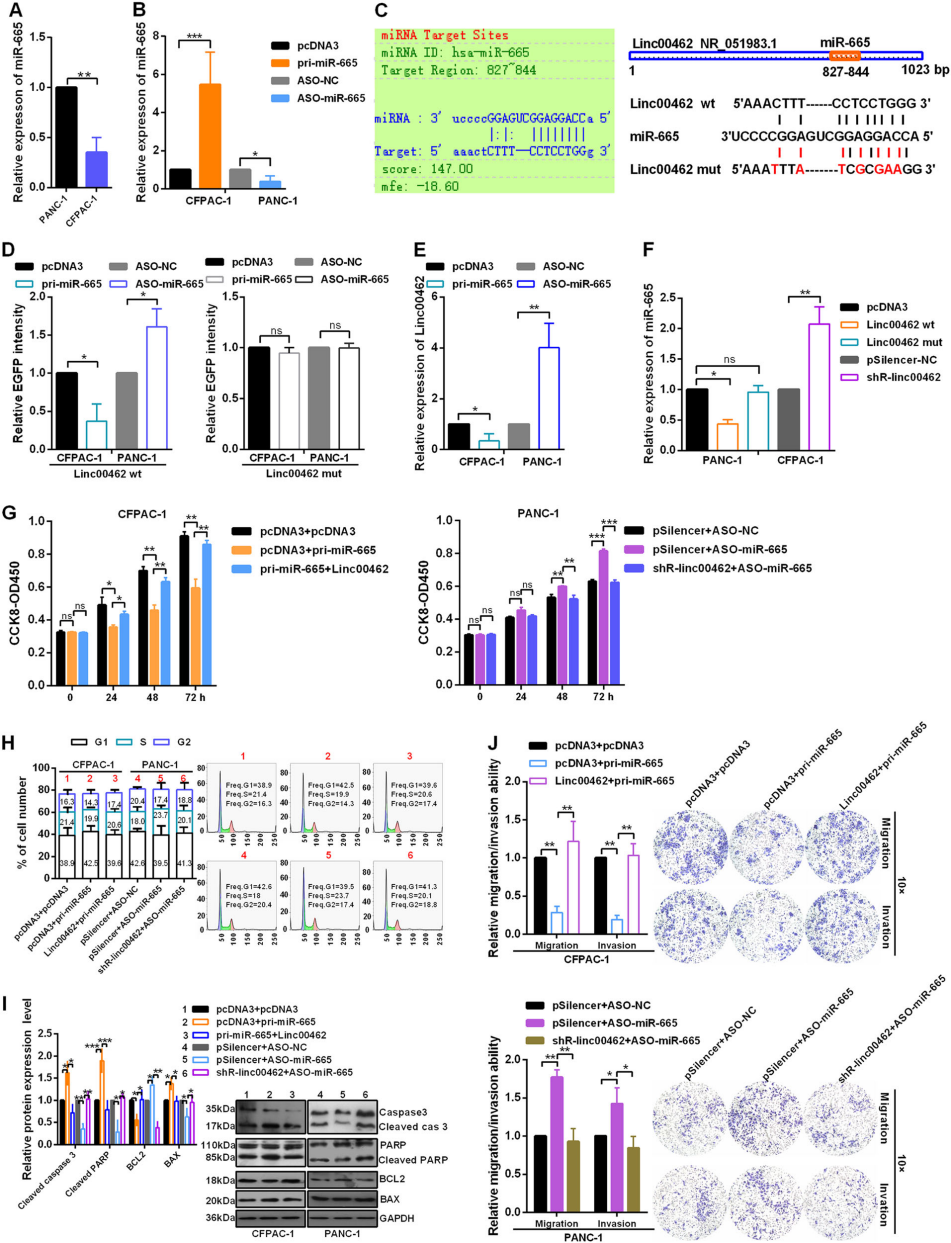
linc00462 interacts with miR-665. **a** The levels of miR-665 in CFPAC-1 and PANC-1 cells were detected by RT-qPCR assay. **b** The efficiency of the indicated plasmids of miR-665 was identified by RT-qPCR assay. **c** The relationship of miR-665 and linc00462 was shown used RegRNA 2.0 and putative and mutant binding sites of miR-665 on linc00462 were shown. **d** EGFP intensity was measured in cells co-transfected with pri-miR-665 or ASO-miR-665 and linc00462 wt or mutant construct. **e** RT-qPCR assay was used to assess linc00462 levels in PC cells in response to altered miR-665 expression. **f** RT-qPCR assay showed the miR-665 levels when the expression of linc00462 was altered. **g** CCK-8 assay showed the cell viability of PC cells treated with the indicated plasmids. **h** Flow cytometry assay showed cell cycle of PC cells treated with the indicated plasmids. **i** Western blot assay showed the related protein levels of cell apoptosis treated with the indicated plasmids. **j** Transwell assay showed the cell migration and invasion transfected with the indicated plasmids. All results performed above are presented as mean ± SD from three independent experiments. *p < 0.05; **p < 0.01; ***p < 0.001, NS not significant

Then, we examine the level of miR-665 in PANC-1 and CFPAC-1 cells and the results showed that the miR-665 level is higher in PANC-1 cells than that in CFPAC-1 cells (Fig. 4a), which negatively was correlated with the expression level of linc00462. As shown in Fig. 4b, RT-qPCR analysis showed that the overexpression and knockdown plasmids of miR-665 were effective (Fig. 4b). Our bioinformatic analysis using RegRNA 2.0 revealed the putative complementary sequences for the seed region of miR-665 in linc00462 (Fig. 4c). To determine whether linc00462 is the target of miR-665, we constructed EGFP reporter vectors containing WT or mutant miR-665 putative binding sites in linc00462. As shown in Fig. 4d, pri-miR-665 decreased and ASO-miR-665 increased the EGFP intensity of cells transfected with the WT reporter vector, while further use of linc00462 mut had no influence (Fig. 4d). Enhanced expression of miR-665 resulted in a significant downregulation of linc00462, while knockdown of miR-665 in PANC-1 cells obviously increased the expression level of linc00462 (Fig. 4e). In addition, ectopic expression of linc00462 significantly decreased the miR-665 levels, while knockdown of linc00462 markedly enhanced the miR-665 levels. However, we did not observe a significant difference in miR-665 level after transfection with linc00462 mut (Fig. 4f). Results from the CCK-8 assay showed overexpression of miR-665 reduced cell proliferation, while co-transfected with linc00462 and pri-miR-665 partly enhanced cell proliferation (Fig. 4g); In contrast, linc00462 knockdown resulted in the opposite effect of cell proliferation. As shown in Fig. 4h, pri-miR-665 markedly increased the number of cells in G1 phase and decreased the number in S + G2 phase, while co-transfection with pri-miR-665 and linc00462 showed that linc00462 abolished the cell cycle-inhibited function of miR-665. Similarly, co-transfection with ASO-miR-665 and shR-linc00462 showed that shR-linc00462 abolished the cell cycle-accelerated function of ASO-miR-665 (Fig. 4h). The expression levels of cleaved caspase 3 and PARP and BAX were increased and the levels of BCL2 were decreased in pri-miR-665 transfected cells, which demonstrate that miR-665 overexpression promoted cell apoptosis (Fig. 4i). The transwell assay showed that migration and invasion of PC cells treated with pri-miR-665 was significantly reduced compared with that of the control. Meanwhile, overexpression of linc00462 partly abolished the reducing effects of on cell migration and invasion. In addition, knockdown of linc00462 abolished the effects of ASO-miR-665 to enhance cell migration and invasion (Fig. 4j).

### Linc00462 increases the expression of the endogenous miR-665 target, TGFBR1 and TGFBR2

**Fig. 5:**
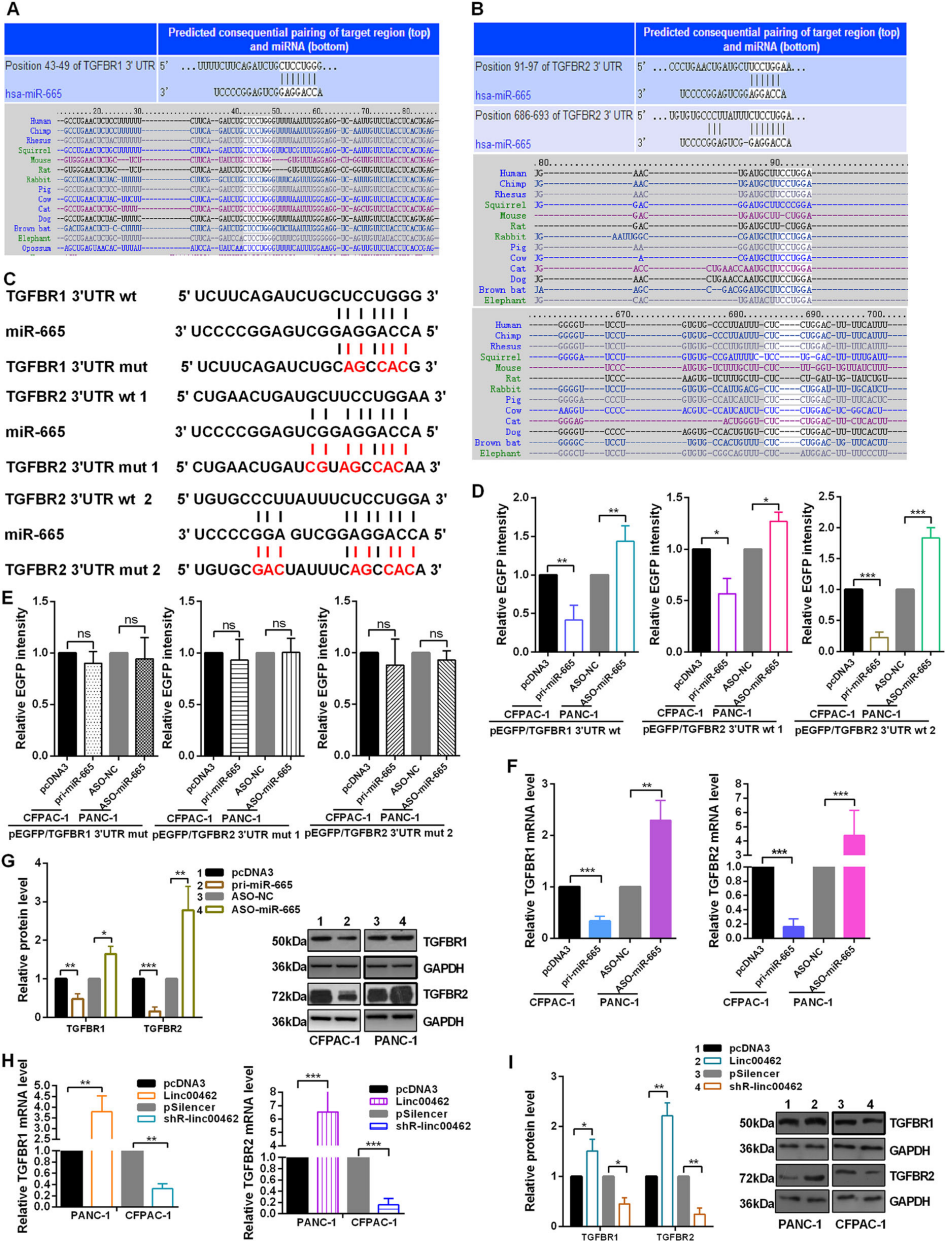
miR-665 directly targets TGFBR1 and TGFBR2. **a**, **b** Targetscan software showed the predicted miR-665 binding site in TGFBR1 and TGFBR2 3′UTRs. **c** The wild type (wt) and mutational (mut) 3′UTRs of TGFBR1 and TGFBR2 mRNAs were shown. **d**, **e** EGFP intensity of PC cells with indicating treatment was determined. **f**, **g** Relative mRNA and protein levels of TGFBR1 and TGFBR2 were shown treated with the miR-665. **h**, **i** Relative mRNA and protein levels of TGFBR1 and TGFBR2 were shown treated with the altered linc00462 using RT-qPCR and western blot assay. All results performed above are presented as mean ± SD from three independent experiments. *p < 0.05; **p < 0.01; ***p < 0.001, NS not significant

Bioinformatics prediction suggested hundreds of candidate targets formiR-665; we chose TGFBR1 and TGFBR2 as putative targets for further study after considering available functional knowledge (Fig. 5a, b). To confirm the direct binding effect of miR-665 to TGFBR1 and TGFBR2 3′UTR, we performed an EGFP reporter assay using EGFP reporter vectors containing either the wild-type 3′UTR or a mutant 3′UTR of TGFBR1 and TGFBR2 (Fig. 5c). Overexpression of miR-665 decreased the EGFP activity of the wild-type 3′UTR of TGFBR1 and TGFBR2 in CFPAC-1 cells, miR-665 knockdown increased the EGFP activity of the wild-type 3′UTR of TGFBR1 and TGFBR2 in PANC-1 cells (Fig. 5d). However, miR-665 overexpression or knockdown did not have any influence on the EGFP activity of the TGFBR1 and TGFBR2 3′UTR mut (Fig. 5e). Accordingly, alteration of miR-665 expression inversely regulated both TGFBR1 and TGFBR2 at mRNA and protein abundance in PC cells (Fig. 5f, g). Ectopic expression of linc00462 increased TGFBR1 and TGFBR2 mRNA and protein levels in PANC-1 cells and that depletion of linc00462 significantly decreased TGFBR1 and TGFBR2 mRNA and protein levels in CFPAC-1 cells (Fig. 5h, i).

### TGFBR1 and TGFBR2 reverses miR-665 overexpression mediated reducing of proliferation, migration, invasion, and EMT

**Fig. 6:**
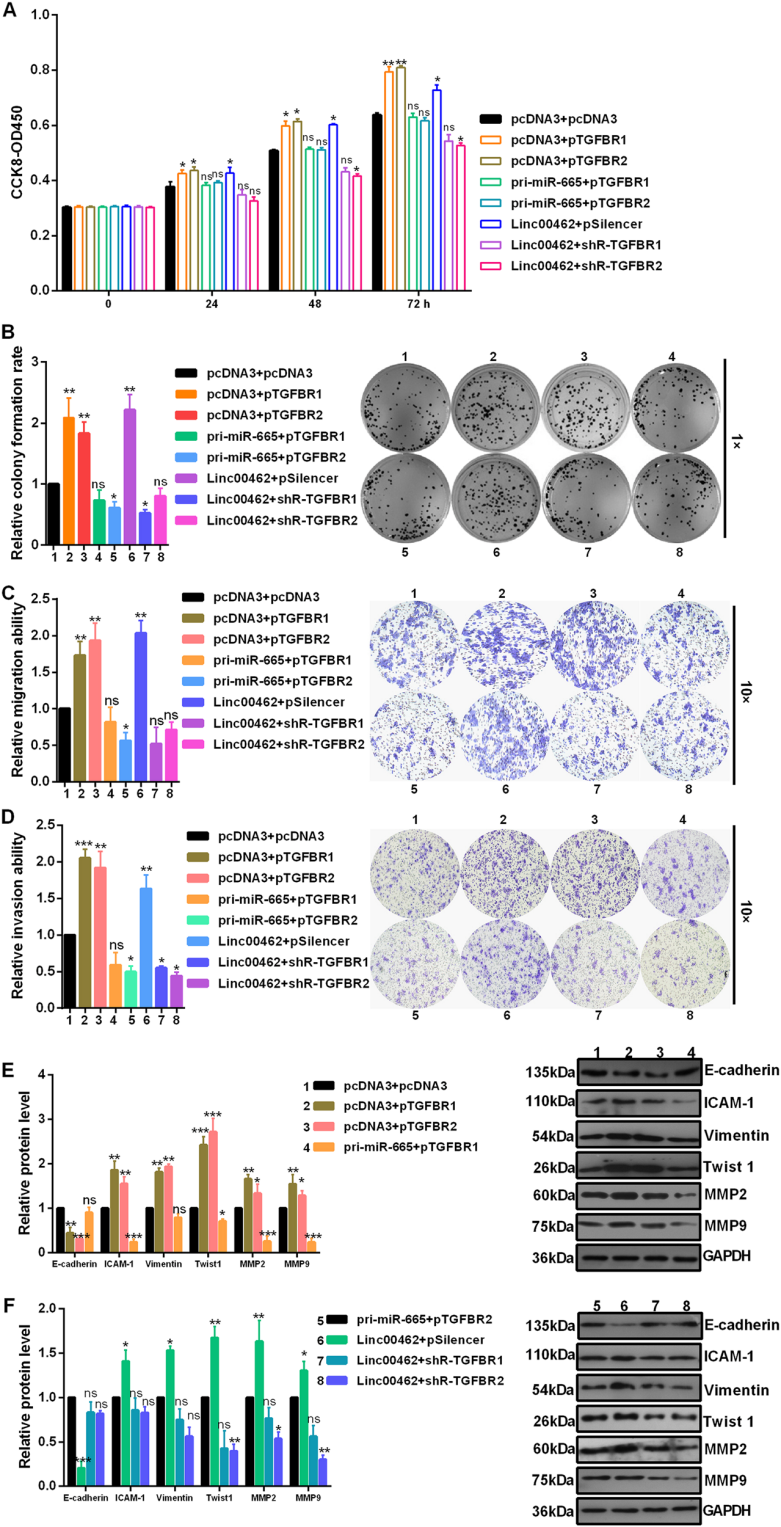
TGFBR1 and TGFBR2 mediated the role of miR-665 and linc00462. Rescue experiments showed cell viability (**a**), colony formation (**b**), cell migration (**c**), cell invasion (**d**), EMT process (**e**, **f**) under the indicated plasmids in CFPAC-1 cells. All results performed above are presented as mean ± SD from three independent experiments. *p < 0.05; **p < 0.01; ***p < 0.001, NS not significant

To further validate the association among linc00462, miR-665 and TGFBR1 and TGFBR2, we then studied the effects of TGFBR1 and TGFBR2 on cell proliferation (Fig. 6a, b), migration (Fig. 6c), invasion (Fig. 6d) and EMT (Fig. 6e). Overexpression of TGFBR1 and TGFBR2 increased cell proliferation, migration, invasion and EMT in CFPAC-1 cells. We observed that after transfected with pri-miR-665, TGFBR1 or TGFBR2 mediated cell malignant behaviors promotion in CFPAC-1 cells was partly rescued. We also observed that after transfected with shR-TGFBR1 or shR-TGFBR2, linc00462 mediated cell malignant behaviors promotion in CFPAC-1 cells was partly rescued (Fig. 6a–f).

### Linc00462 promotes and miR-665 inhibits the malignant phenotype of PC by regulating the TGFBR1 and TGFBR2 induced SMAD2/SMAD3 signaling pathway

**Fig. 7:**
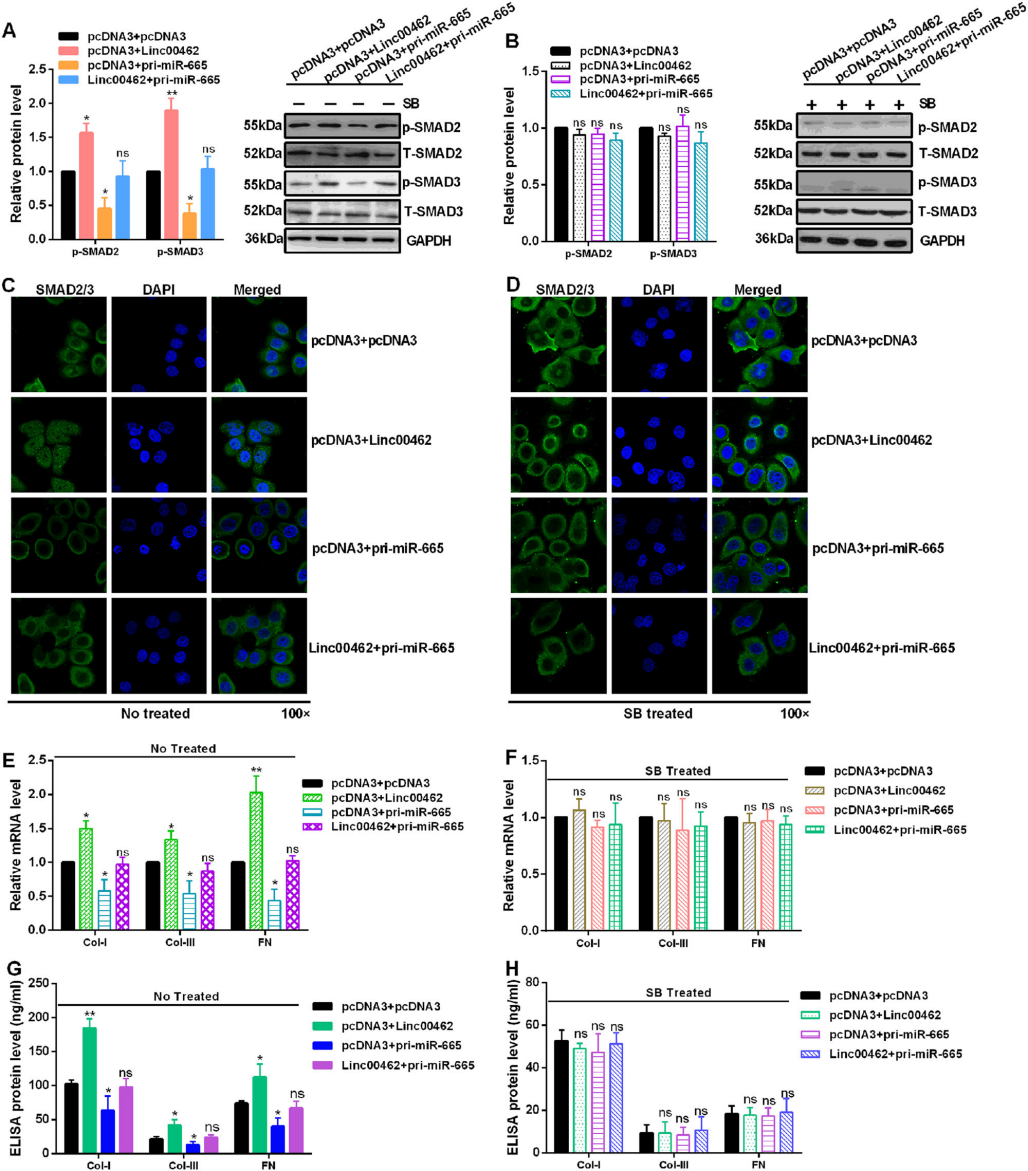
Linc00462 and miR-665 regulate the SMAD2/3 pathway. **a** The indicated protein levels were detected by western blot under the non-treated condition in CFPAC-1 cells. **b** The indicated protein levels were detected by western blot treated with the TGFBR1/2 inhibitor SB431542 for 2 h in CFPAC-1 cells. **c**, **d** The distribution of SMAD2/3 was shown by IF assay treated with the TGFBR1/2 inhibitor SB431542 for 2 h or not in CFPAC-1 cells. **e**, **f** Relative mRNA levels of collagen 1, collagen 3, and fibronectin were detected by RT-qPCR assay treated with the TGFBR1/2 inhibitor SB431542 for 2 h or not in CFPAC-1 cells. **g**, **h** ELISA assay showed the levels of collagen 1, collagen 3, and fibronectin treated with the TGFBR1/2 inhibitor SB431542 for 2 h or not in CFPAC-1 cells. All results performed above are presented as mean ± SD from three independent experiments. *p < 0.05; **p < 0.01; ***p < 0.001, NS not significant

Some papers reported that activated TGF-β type I receptor subsequently induces the recruitment and phosphorylation of the receptor-regulated SMADs, SMAD2 and SMAD3 to form heteromeric complexes with the common mediator SMAD4. The SMAD complexes transmited the signals of TGF-β from cell surface to nucleus and bind to the target genes to exhibit the regulated role^23–25^. Our data showed that linc00462 overexpression significantly increased the expression levels of p-SMAD2 and p-SMAD3. miR-665 overexpression significantly decreased the expression levels of p-SMAD2 and p-SMAD3. However, co-transfected with linc00462 and miR-665 reversed the up-regulation induced by linc00462 overexpression or down-regulation induced by miR-665 overexpression of p-SMAD2 and p-SMAD3 (Fig. 7a). To determine whether linc00462 activation of SMAD2 and SMAD3 require TGFBR1 and TGFBR2 expression, cells were treated for 2 h the TGFBR1/2 inhibitor SB431542. The results showed that alteration of linc00462, miR-665 or linc00462 and miR-665 has no difference on the expression level of p-SMAD2 and p-SMAD3, which indicate TGFBR1 and TGFBR2 was indispensable for activation of SMAD2/SMAD3 signaling pathway (Fig. 7b). To investigate the transformation of SMAD2/3 from cell surface to nucleus, IF assay was performed. The results showed that overexpression of linc00462 increased the nuclear distribution of SMAD2/3. Overexpression of miR-665 decreased the nuclear distribution of SMAD2/3. And co-transfected with linc00462 and pri-miR-665 partly rescued the effects of linc00462 or miR-665 in CFPAC-1 cells (Fig. 7c). However, no significant difference about the nuclear distribution of SMAD2/3 was observed among the respective groups in cells treated with SB431542 (Fig. 7d). Furthermore, treatment with linc00462 markedly enhanced the expression of collagen 1, collagen 3 and fibronectin mRNA level by RT-qPCR assay and protein level by ELISA assay in CFPAC-1 cells. Co-transfected with linc00462 and pri-miR-665 partly rescued the effects of linc00462 or miR-665 on collagen 1, collagen 3, and fibronectin levels in CFPAC-1 cells (Fig. 7e–g). In contrast, no significant difference was observed about the expression level of collagen 1, collagen 3 and fibronectin in cells treated with SB431542 (Fig. 7f–h).

## Discussion

LncRNAs have emerged as a new layer of gene regulation in many diseases, at the transcriptional and posttranscriptional level of the gene regulatory network^26–28^. Up to now, emerging evidences have focused on the role and the function of lncRNAs in PC progression^29–31^. Linc00462 was located on chromosome 13 and was approximately 1023 nt in length, which was upregulated in HCC tissues compared with normal tissues^18^. However, no relevant report about the relationship between linc00462 and the progression of PC is reported now.

In this study, we reported that linc00462, which could be activated by OSM, was significantly upregulated in PC tissues and cell lines compared with the control groups. Its expression level was significantly correlated with tumor size, tumor differentiation, TNM stage, and distant metastasis. Furthermore, overexpression of linc00462 significantly promoted cell proliferation, migration, invasion and cell cycle process and inhibited cell apoptosis and cell adhesion by inducing EMT in vitro functional assays. Moreover, we demonstrated that linc00462 plays an oncogenic role in PC by promoting tumor growth and metastasis using the vivo animal model. Taken together, these data indicate that linc00462 functions as an oncogene and promotes PC malignant progression. Linc00462 may represent a novel therapeutic target for PC treatment.

However, the molecular mechanisms by which linc00462 promote cell proliferation, cell migration, cell invasion and tumor metastasis remain unclear. Previous studies identified that lncRNAs and miRNAs may communicate with and co-regulate each other via multiple mechanisms, the competing endogenous RNA (ceRNA) hypothesis included^32,33^. To investigate whether linc00462 regulate PC progression through the ceRNA hypothesis, we found binding sites between linc000462 and miR-665, and thus carried out the follow-up study using bioinformatics analysis. In addition, EGFP reporter assay and RT-qPCR assay showed that linc00462 suppressed the expression of miR-665 via sponging miR-665 in the PC cells. MiR-665 plays a suppressive role in osteosarcoma^34^, inflammatory bowel disease^35^, prostate cancer^36^, and severe heart failure^37^. However, the function of miR-665 in PC remains unknown. We reported that overexpression of miR-665 in PC cells inhibited cell proliferation, cell cycle process, cell migration and cell invasion, and promoted cell apoptosis. In addition, bioinformatics tools were used to identify the potential downstream targets of miR-665. The analysis suggested that TGFBR1 and TGFBR2 might be the downstream targets of miR-665.

Wang reported that miR-130a-3p might play a critical role in negatively regulating HSC activation and proliferation in the progression of nonalcoholic fibrosing steatohepatitis by directly targeting TGFBR1 and TGFBR2 via the TGF-β/SMAD signaling pathway^38^. Wang et al. reported that miR-133a may suppress cell invasion by targeting TGFBR1 in non-small cell lung carcinoma^39^. Harazono et al. reported overexpression of miR-655 not only induced the up-regulation of E-cadherin and downregulation of typical EMT-inducers but also suppressed migration and invasion of mesenchymal-like cancer cells accompanied by a morphological shift toward the epithelial phenotype by targeting ZEB1 and TGFBR2^40^. However, the role of TGFBR1 and TGFBR2 in PC is unclear. In this study, we showed that overexpression of TGFBR1 and TGFBR2 promoted cell proliferation, cell migration, cell invasion and EMT in PC cells. Further exploration showed that linc00462 shares the same response elements for miR-665 with TGFBR1 and TGFBR2. Enhanced expression of linc00462 increased the mRNA and protein levels of TGFBR1 and TGFBR2.

Smads can integrate multiple signaling pathways and directly regulate the expression of target genes in TGF-β-activated cells^41^. Binding of TGF-β to its receptor leads to activation of the transcription factors, Smad2/3, and then translocate into the nucleus where the factors can induce transcription of target genes^42^. Our results showed linc00462 overexpression significantly increased but miR-665 overexpression obviously decreased the expression levels of p-SMAD2 and p-SMAD3 and the nuclear distribution of SMAD2/3. However, alteration of linc00462, miR-665 or linc00462 and miR-665 has no difference on the expression levels of p-SMAD2 and p-SMAD3 and the nuclear distribution of SMAD2/3 treated with SB431542.

Considering the importance of the proposed mechanisms of action, we turned our attention to the SMAD2/3 pathways involved in stimulation of collagen expression. Thus, we detected the expression levels of collagen 1, collagen 3 and fibronectin by RT-qPCR and ELISA assay, and the results showed that linc00462 overexpression markedly enhanced and miR-665 overexpression obviously attenuated the expression of collagen 1, collagen 3, and fibronectin at mRNA and protein levels. However, no significant difference was observed about the expression level of collagen 1, collagen 3, and fibronectin in cells treated with SB431542.

**Fig. 8:**
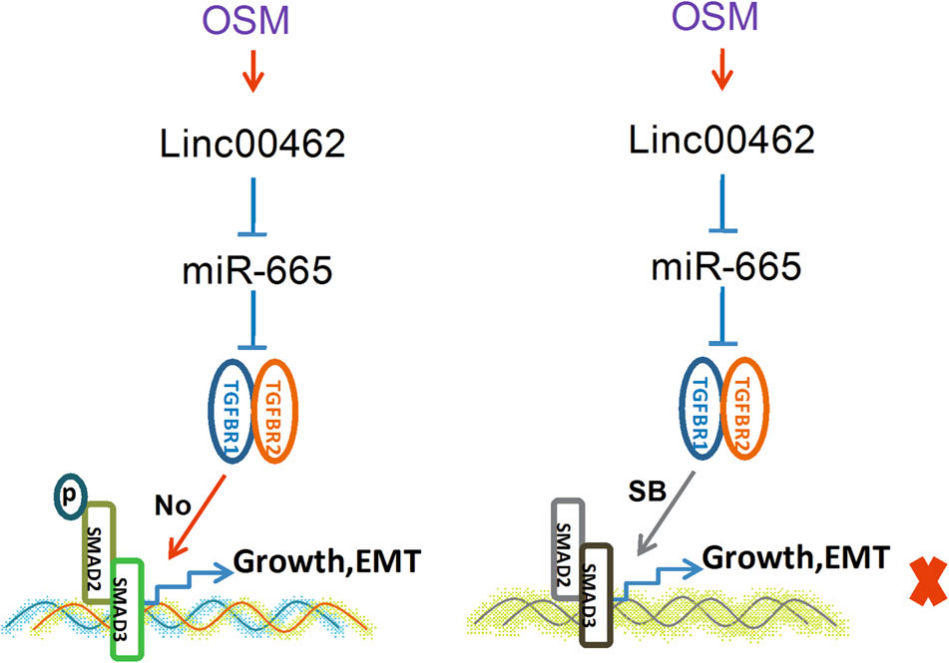
The model of linc00462 regulation in PC

In conclusion, OSM-mediated linc00462 via miR-665/TGFBR1-TGFBR2/smad2/3 pathway was a crucial event during the cell proliferation, cell migration and invasion and tumor metastasis in PC (Fig. 8). Our findings could be useful for developing novel therapeutics against metastatic PC.

## Methods and materials

### Patients

Pancreatic samples were obtained from the Department of Hepatopacreatobiliary, Affiliated Hospital of Qingdao University. All patients did not receive chemotherapy or radiotherapy before the surgery. All tissue samples were derived from untreated patients undergoing surgery, and were frozen in liquid nitrogen immediately and stored at −80 °C. The written informed consents were obtained from all enrolled patients, and all relevant investigations were performed according to the principles of the declaration of Helsinki. The study was approved by the ethical review committees of Affiliated Hospital of Qingdao University.

### Cell lines

Human pancreatic cancer cell lines SW1990, BxPC-3, PANC-1, AsPC-1, and CFPAC-1 were obtained from the American Type Culture Collection. Normal pancreatic cells HPDE6-C7 were purchased from Chinese Academy of Sciences (Shanghai, China). All cell lines were cultured in 37 °C in a 5% CO2 humidified in DMEM or RPMI 1640 (Invitrogen, Carlsbad, CA) with 10% fetal bovine serum (BI, Carlsbad, CA), 100 U/ml penicillin, and 100 μg/ml streptomycin (Sigma-Aldrich, St. Louis, MO, USA).

### Vector construction

Expression vectors encoding linc00462, TGFBR1 (NM_004612.3) and TGFBR2 (NM_001024847.2) were constructed by cloning the open reading frames into the pcDNA3 (Ambion, Austin, TX, USA) vector. For miR-665 expression vector (pri-miR-665), 400 bp containing miR-665 encoded region was amplified from genomic DNA and cloned into the pcDNA3 vector. The 2′-O-methyl-modified miR-665 antisense oligonucleotide (ASO-miR-665) was commercially synthesized as an inhibitor of miR-665. The shR-linc00462, shR-TGFBR1 and shR-TGFBR2 plasmids expressing siRNA were constructed by annealing double-strand hairpin cDNA and inserting it into the pSilencer 2.1-U6 neo vector (Ambion, Austin, TX, USA). All primers for PCR amplification are provided in Supplement Table 2.

**Table 2:** The primers used in this study

RT-qPCR primers	Sequences (5′-3′)
Linc00462-qPCR-forward	ACTAGGTCCTTCTGGTGTT
Linc00462-qPCR-reverse	GTAAAACTTGCTGCTGATG
U6-RT	GTCGTATCCAGTGCAGGGTCCGA
	GGTGCACTGGATACGACAAAATATGG
U6-qPCR-forward	TGCGGGTGCTCGCTTCGGCAGC
Reverse-miRNAs	CCAGTGCAGGGTCCGAGGT
β-actin-qPCR-forward	CGTGACATTAAGGAGAAGCTG
β-actin-qPCR-reverse	CTAGAAGCATTTGCGGTGGAC
Oligo dT	TTTTTTTTTTTTTTTTTT
miR-665-RT	GTCGTATCCAGTGCAGGGTCCG
	AGGTGCACTGGATACGACAGGGGC
miR-665-qPCR-forward	TGCGGACCAGGAGGCTGAG
TGFBR2-qPCR-forward	TCTGGGCTCCTGATTGCT
TGFBR2-qPCR-reverse	TGAGGCAGCTTTGTAAGT
TGFBR1-qPCR-forward	AAAACATTATCGCAACTCAG
TGFBR1-qPCR-reverse	CACAGAAAGGACCCACAT
COI-I-qPCR-forward	GGCAACAGCCGCTTCACCT
COI-I-qPCR-reverse	AGGGAGCCAGGTTGGGATG
COI-III-qPCR-forward	CCCTGTCTGCTTCCTGTA
COI-III-qPCR-reverse	GCAACCATCCTCCAGAAC
FN-qPCR-forward	TGAACCCAGTCCCGAAGG
FN-qPCR-reverse	AACTCCCAGGGTGATGCT
*Plasmids primers*	
linc00462-S	CGGGGTACCCCCAGTCTCGGATAAGTC
linc00462-AS	CCGCTCGAGGTCCTCGAACATTCCACT
miR-665-S	CGCGGATCCCTTTGTGCCCAGGGTGGA
miR-665-AS	GAATTCAGACAGAGGTCCAAGAACCAG
TGFBR1-S	CCCAAGCTTGAATCCATGGAGGCGGCGGTCGCTGCT
TGFBR1-AS	CTCGAGTTACATTTTGATGCCTTCCTGTTGA
TGFBR2-S	CCCAAGCTTGAATCCATGGGTCGGGGGCTGCTCAGG
TGFBR2-AS	CTCGAGTTA CTATTTGGTAGTGTTTAGGGAGCCG
ASO-NC	CAGUACUGUAGUGUAGUACTT
ASO-miR-665	AGGGGCCUCAGCCUCCUGGU
shR-linc00462-S	CCGGAAGCACAGTGGTCTAAAAGTA
	CTCGAGTACTTTTAGACCACTGTGCTTTTTTTG
shR-linc00462-AS	CAAAAAAAGCACAGTGGTCTAAAAGT
	ACTCGAGTACTTTTAGACCACTGTGCTTCCGG
shR-TGFBR1-S	GATCCGGGCTGACAGCTTTGCGAATTAA
	CTCGAGTTAATTCGCAAAGCTGTCAGCTTTTTA
shR-TGFBR1-AS	AGCTTCAAAAAGGGCTGACAGCTTTGCGAATT
	AACTCGAGTTAATTCGCAAAGCTGTCAGCG
shR-TGFBR2-S	GATCCGGGACCTCAAGAGCTCCAATAT
	CCTCGAGGATATTGGAGCTCTTGAGGTCTTTTTGA
shR-TGFBR2-AS	AGCTTCAAAAAGGGACCTCAAGAGCTCCA
	ATATCCTCGAGGATATTGGAGCTCTTGAGGTCG

### Quantitative real time PCR analyses

The total RNA was extracted from the tissues or cultured cells using Trizol reagent (Invitrogen, Carlsbad, CA, USA), according to the manufacturer’s instructions. The quality and integrity of acquired RNA was evaluated by Nanodrop2000c and gel electrophoresis, respectively. RT-qPCR was performed with Prime Script reagent RT Kit and SYBR Prime Script RT-PCR Kits (Takara, Dalian, China) based on the manufacturer’s instructions. All real-time PCR reactions were performed using a 7500 Fast Real Time PCR System (Applied Biosystems). The target gene expression level was calculated with the 2^−ΔΔCt^ method, which was normalized to β-catin mRNA. The relative fold changes were calculated using the 2^-ΔΔCt^ method with their corresponding inner control genes. The primers used in this study were shown in Table 2.

### CCK-8 assay

PC cells were seeded at a density of 6000 cells per well in 96-well plates. The PANC-1 and CFPAC-1 cells were transfected with the indicated plasmids. Cell viability at 0, 24, 48, and 72 h post-transfection was determined by Cell Counting Kit-8 (CCK-8; KeyGen Biotech) according to the manufacturer’s protocol. The absorbance values at 450 nm were measured via the Quant Microplate Spectrophotometer (BioTek, Winooski, VT).

### Colony formation assay

For the colony formation assay, cells were seeded at a low density (500 cells per plate) and allowed to grow until visible colonies appeared. Culture medium was replaced every 72 h. The cells were then stained with common crystal violet dye, and colonies containing more than 50 cells were counted.

### Prediction of linc00462 and miR-665 targets

The correlation of linc00462 and miRNAs was predicted by RegRNA 2.0. The hypothetical targets of miR-665 were predicted using Targetscan 7.1, RNAhybrid, and microRNA.org.

### EGFP reporter assay

The 3′UTRs of TGFBR1 and TGFBR2 that contain the miR-665 binding sites and mutant 3′UTR fragments with mutant miR-665 binding sites were obtained by annealing double-strand DNA and inserting it into the pcDNA3/EGFP (Tianjin Saierbio, Tianjin, China) vector. All primers for PCR amplification are provided in Table 2. The EGFP reporter plasmids with TGFBR1 and TGFBR2 3′UTR or 3′UTR-mut were transfected into PANC-1 and CFPAC-1 cells with Lipofectamine^TM^ 2000 reagent (Invitrogen, Carlsbad, CA), and RFP expressing plasmid was integrated as a transfection efficiency control. Cells were lysed 48 h post-transfection, and the intensities of EGFP and RFP fluorescence were determined with a spectrophotometer.

### Cell-matrix adhesion assay

96-well plates were coated overnight with 10 mg/ml fibronectin (Solarbio, Shanghai, China) at 4 °C. Cells were seeded on the 96-well plates at a density of 5 × 10^3^/well, allowed to adhere at 37 °C for at 10, 30, 60 min, and were then washed three times with 1 × PBS. The cells were fixed with 4% (v/v) paraformaldehyde, stained with 0.5% (w/v) crystal violet for 10 min, and then lysed with 30% (v/v) glacial acetic acid for 10 min; absorbance at 620 nm was then measured.

### Western blot assay

Cell extracts were cleaned with 1× PBS buffer, prepared with RIPA lysis buffer, and analyzed by immunoblotting. Antibodies of cleaved caspase 3, cleaved PARP, BCL2, BAX, E-cadherin, ICAM-1, Vimentin, Twist 1, MMP2, MMP9, GAPDH, SMAD2, SMAD3, p-SMAD2, p-SMAD3, TGFBR1, and TGFBR2 were purchased from CST (Cell Signaling Technology, Danvers, MA, USA) and the secondary goat anti-rabbit antibody was obtained from Santa (Cruz Biotechnology, Dallas, TX, USA). LabWorks^TM^ Image Acquisition and Analysis Software (UVP, Upland, CA) were used to quantify band intensities.

### Cell cycle and apoptosis flow cytometry analyses

At 48 h after transfection, transfected PC cells were harvested by trypsinization and resuspended in cold phosphate-buffered saline for analysis. For the analysis of cell cycle, cells stained with PI according to the manufacturer’s manual. The rate of cell apoptosis was detected using an Annexin V-FITC/PI apoptosis detection kit (BD Biosciences, San Jose, CA, USA) according to the manufacturer’s instructions.

### Cell invasion assay

The Boyden chambers with 8 μm pores (Corning, NY, USA) were used to evaluate cell motility. The transwell membrane (filter) was pre-coated with 30 μl of matrigel: PBS (1:3) and incubated for 48 h. The transfected 1 × 10^4^ cells were resuspended in 100 μl serum-free medium and then transferred to the upper chambers. Approximately 600 μl medium with 10% serum was added to the lower chamber. After incubation for 24 h, the transwell membrane was fixed with methanol, stained with crystal violet, and then counted under a light microscope.

### Immunofluorescence

Cells seeded on glass coverslips in 24-well plates were fixed in 4% formaldehyde solution and permeabilized with 0.05% Triton X-100. Cells were blocked with 10% bovine serum albumin for 15 min and incubated with primary antibody (p-SMAD2/3, Cell Signaling, Beverly, MA.) at room temperature for 4 h, followed by incubation with FITC-conjugated secondary antibodies (FITC, Invitrogen, Carlsbad, CA) for 45 min, and then stained with DAPI. Finally, images were taken under a confocal microscope (OLYMPUS, Tokyo, Japan).

### In vivo experiments

BALB/c nude mice, were purchased from the Shanghai Experimental Animal Center-Chinese Academy of Sciences (Shanghai, China), were randomly assigned to 2 groups (*n* = 6), 2 × 10^7^ cells (PANC-1-pcDNA3 or PANC-1-linc00462) resuspended in 25 µl of DMEM and mixed with 25 µl of Matrigel (1 mg/ml) were injected into the tail vein of mice. After 6–8 weeks, the mice were euthanized, and the spleen, colorectum, lung and liver were removed for pathological examination and stained with H&E. In heterograft experiment, lentivirus (1 × 10^7^) transduced PANC-1 cells were subcutaneously injected into the right armpit of BALB/c nude mice. The weight of the mice and the diameters of tumors were measured.

### Statistical analysis

All analyses were performed using SPSS 19 for Windows (SPSS Inc., Chicago, IL, USA) and GraphPad Prism 5 for Windows (GraphPad Software Inc., San Diego, CA, USA). For comparisons of two treatment groups, the Student *t*-test was used. For comparisons of three or more groups, one-way ANOVA was used with the Bonferroni post-hoc test for comparison of two selected treatment groups; the Dunnett post-hoc test was used for comparisons of the other treatment groups with the corresponding controls. Data are presented as means ± standard deviation (SD) or medians with ranges. Statistical analyses were performed using Student’s *t*-tests. A *p*-value less than 0.05 are considered significant. *p < 0.05; **p < 0.01, ***p < 0.001.

## References

[1] Siegel RL, Miller KD, Jemal A (2016). Cancer statistics, 2016. Cancer J. Clin..

[2] Bianconi D (2017). Biochemical and genetic predictors of overall survival in patients with metastatic pancreatic cancer treated with capecitabine and nab-paclitaxel. Sci. Rep..

[3] Binenbaum Y, Na’ara S, Gil Z (2015). Gemcitabine resistance in pancreatic ductal adenocarcinoma. Drug Resist. Update.

[4] Zhang JQ (2018). MicroRNA-300 promotes apoptosis and inhibits proliferation, migration, invasion and epithelial-mesenchymal transition via the Wnt/beta-catenin signaling pathway by targeting CUL4B in pancreatic cancer cells. J. Cell. Biochem..

[5] Krebs AM (2017). The EMT-activator Zeb1 is a key factor for cell plasticity and promotes metastasis in pancreatic cancer. Nat. Cell Biol..

[6] Aiello NM (2017). Upholding a role for EMT in pancreatic cancer metastasis. Nature.

[7] Ponting CP, Oliver PL, Reik W (2009). Evolution and functions of long noncoding RNAs. Cell.

[8] Frith MC (2006). Discrimination of non-protein-coding transcripts from protein-coding mRNA. RNA Biol..

[9] Guttman M (2011). lincRNAs act in the circuitry controlling pluripotency and differentiation. Nature.

[10] Niu Y (2017). Long non-coding RNA TUG1 is involved in cell growth and chemoresistance of small cell lung cancer by regulating LIMK2b via EZH2. Mol. Cancer.

[11] Lu W (2017). Long non-coding RNA linc00673 regulated non-small cell lung cancer proliferation, migration, invasion and epithelial mesenchymal transition by sponging miR-150-5p. Mol. Cancer.

[12] Li Z (2015). The long non-coding RNA HOTTIP promotes progression and gemcitabine resistance by regulating HOXA13 in pancreatic cancer. J. Transl. Med..

[13] Zhao L (2017). LncRNA-PVT1 promotes pancreatic cancer cells proliferation and migration through acting as a molecular sponge to regulate miR-448. J. Cell. Physiol..

[14] Sun YW (2014). A novel long non-coding RNA ENST00000480739 suppresses tumour cell invasion by regulating OS-9 and HIF-1alpha in pancreatic ductal adenocarcinoma. Br. J. Cancer.

[15] Peng W, Gao W, Feng J (2014). Long noncoding RNA HULC is a novel biomarker of poor prognosis in patients with pancreatic cancer. Med. Oncol..

[16] Qin CF, Zhao FL (2017). Long non-coding RNA TUG1 can promote proliferation and migration of pancreatic cancer via EMT pathway. Eur. Rev. Med. Pharmacol. Sci..

[17] Cai H (2017). LncRNA HOTAIR acts a competing endogenous RNA to control the expression of notch3 via sponging miR-613 in pancreatic cancer. Oncotarget.

[18] Gong J (2017). Long noncoding RNA linc00462 promotes hepatocellular carcinoma progression. Biomed. Pharmacother..

[19] Bartonicek N, Maag JL, Dinger ME (2016). Long noncoding RNAs in cancer: mechanisms of action and technological advancements. Mol. Cancer.

[20] Salmena L, Poliseno L, Tay Y, Kats L, Pandolfi PP (2011). A ceRNA hypothesis: the Rosetta Stone of a hidden RNA language?. Cell.

[21] Wei W, Liu Y, Lu Y, Yang B, Tang L (2017). LncRNA XIST promotes pancreatic cancer proliferation through miR-133a/EGFR. J. Cell. Biochem..

[22] Smigiel JM, Parameswaran N, Jackson MW (2017). Potent EMT and CSC phenotypes are induced by oncostatin-M in pancreatic cancer. Mol. Cancer Res..

[23] Zhang M (2018). S100A11 promotes TGF-β1-induced epithelial-mesenchymal transition through SMAD2/3 signaling pathway in intrahepatic cholangiocarcinoma. Future Oncol..

[24] Saitoh M (2015). Epithelial-mesenchymal transition is regulated at post-transcriptional levels by transforming growth factor-beta signaling during tumor progression. Cancer Sci..

[25] Shi Y, Massague J (2003). Mechanisms of TGF-beta signaling from cell membrane to the nucleus. Cell.

[26] Hao NB, He YF, Li XQ, Wang K, Wang RL (2017). The role of miRNA and lncRNA in gastric cancer. Oncotarget.

[27] Bolha L, Ravnik-Glavac M, Glavac D (2017). Long noncoding RNAs as biomarkers in cancer. Dis. Markers.

[28] Bhan A, Soleimani M, Mandal SS (2017). Long noncoding RNA and cancer: a new paradigm. Cancer Res..

[29] Peng JF, Zhuang YY, Huang FT, Zhang SN (2016). Noncoding RNAs and pancreatic cancer. World J. Gastroenterol..

[30] Pan Y (2016). The emerging roles of long noncoding RNA ROR (lincRNA-ROR) and its possible mechanisms in human cancers. Cell. Physiol. Biochem..

[31] Huang X (2016). LncRNAs in pancreatic cancer. Oncotarget.

[32] Yoon JH, Abdelmohsen K, Gorospe M (2014). Functional interactions among microRNAs and long noncoding RNAs. Semin. Cell Dev. Biol..

[33] Qu J, Li M, Zhong W, Hu C (2015). Competing endogenous RNA in cancer: a new pattern of gene expression regulation. Int. J. Clin. Exp. Med..

[34] Dong C (2016). MicroRNA-665 suppressed the invasion and metastasis of osteosarcoma by directly inhibiting RAB23. Am. J. Transl. Res..

[35] Li M (2017). Upregulation of miR-665 promotes apoptosis and colitis in inflammatory bowel disease by repressing the endoplasmic reticulum stress components XBP1 and ORMDL3. Cell Death Dis..

[36] Sadeghi M (2016). MicroRNA and transcription factor gene regulatory network analysis reveals key regulatory elements associated with prostate cancer progression. PLoS One.

[37] Mohnle P (2014). MicroRNA-665 is involved in the regulation of the expression of the cardioprotective cannabinoid receptor CB2 in patients with severe heart failure. Biochem. Biophys. Res. Commun..

[38] Wang Y (2017). MiR-130a-3p attenuates activation and induces apoptosis of hepatic stellate cells in nonalcoholic fibrosing steatohepatitis by directly targeting TGFBR1 and TGFBR2. Cell Death Dis..

[39] Wang LK (2014). MicroRNA-133a suppresses multiple oncogenic membrane receptors and cell invasion in non-small cell lung carcinoma. PLoS One.

[40] Harazono Y (2013). miR-655 Is an EMT-suppressive microRNA targeting ZEB1 and TGFBR2. PLoS One.

[41] Kakonen SM (2002). Transforming growth factor-beta stimulates parathyroid hormone-related protein and osteolytic metastases via Smad and mitogen-activated protein kinase signaling pathways. J. Biol. Chem..

[42] Tsang KJ, Tsang D, Brown TN, Crowe DL (2002). A novel dominant negative Smad2 mutation in a TGFbeta resistant human carcinoma cell line. Anticancer Res..

